# The relationship between screen-based sedentary behaviors and symptoms of depression and anxiety in youth: a systematic review of moderating variables

**DOI:** 10.1186/s12889-020-08572-1

**Published:** 2020-04-10

**Authors:** Jennifer Zink, Britni R. Belcher, Kellie Imm, Adam M. Leventhal

**Affiliations:** 1 Department of Preventive Medicine, Keck School of Medicine of University of Southern California, 2001 North Soto Street, Third Floor, California, Los Angeles 90032 USA; 2 Department of Psychology, University of Southern California, Los Angeles, USA

**Keywords:** Emotional health, Inactivity, Screen time, Digital media, Children, Adolescents

## Abstract

**Background:**

To elucidate the populations and conditions where screen-based sedentary behaviors (SB) and internalizing symptoms are coupled, this review synthesized the evidence for factors that may moderate the associations between screen-based SB, depressive symptoms, and anxiety symptoms among youth.

**Methods:**

Two independent researchers conducted a systematic literature search of the Medline, psycINFO, and Scopus electronic databases in late 2018 for observational studies assessing moderators of the association between screen-based SB and depressive and anxiety symptoms. Studies among children and adolescents were eligible if moderation was assessed by statistical test (interaction) or stratification; and a narrative synthesis of eligible studies was conducted in accordance with PRISMA guidelines.

**Results:**

Seventy empirical studies (46 cross-sectional, 19 longitudinal, and 5 both) of 13 different moderating variables of screen-based SB-internalizing symptom associations met the eligibility criteria. Of these, 40 studies were of depressive symptoms, 2 were of anxiety symptoms, and 28 studies assessed symptoms of both. The most consistent evidence of moderation was for screen-type, such that TV viewing was not as strongly associated with internalizing symptoms compared to other forms of screen-based SB. There was also inconsistent evidence for physical activity buffering screen-based SB-internalizing symptom associations and for female sex amplifying screen-based SB-internalizing symptom associations. In general, the body of evidence for anxiety symptoms was more limited than that for depressive symptoms, and were therefore more inconsistent.

**Conclusions:**

Screen-type, physical activity, and sex may influence the magnitude of screen-based SB-internalizing symptom coupling; highlighting potential sources of heterogeneity of screen-based SB-internalizing symptom associations. Additional studies aimed at understanding potential mechanistic explanations for the above moderators are needed prior to the development of tailored intervention strategies designed to decouple screen-based SB and internalizing symptoms among youth.

## Introduction

Screen-based sedentary behaviors (SB) such as television viewing and computer use have become ubiquitous in children and adolescents, in part because of the increasing availability of youth-friendly digital entertainment [1]. Even more concerning, the prevalence of screen-based SB increases across childhood and adolescence [2]. Because screen-based SB are modifiable behaviors that strongly predict future levels of screen-based SB [3] and consequently adverse health-outcomes in adulthood [4], understanding the determinants, correlates, and consequences of screen-based SB among youth is critical for informing preventive interventions that may benefit health throughout the lifespan.

Paralleling the rising rates of screen-based SB, the adolescent developmental period is also a high-risk period for the onset of internalizing symptoms and disorders [5]. Recent national U.S. estimates indicate the lifetime prevalence of depressive or anxiety disorders in adolescents are 11.7 and 31.9%, respectively [6], but the incidence of subclinical depression and anxiety levels is much higher [7, 8]. Depression and anxiety in youth – even at levels below the threshold of a psychiatric diagnosis – increase risk for suicide, substance misuse, obesity [9, 10], poorer social development, and worse academic performance [11].

Previous systematic reviews and meta-analyses have provided evidence that screen-based SB and internalizing symptoms may be associated with one another among youth [12–14]. It is believed that psychosocial mechanisms play a role in linking screen-based SB and internalizing symptoms. For example, the social withdrawal theory postulates that engaging in screen-based SB can lead to social isolation, which may increase risk for internalizing symptoms [15]. While the abovementioned systematic reviews and meta-analyses were critical for identifying potentially positive associations between screen-based SB and internalizing symptoms, they also highlighted inconsistencies in the findings across the individual studies [12, 14]. The heterogeneity of findings may, in part, be a result of unexplored moderators of the association between screen-based SB and internalizing symptoms.

A current gap in the literature is the lack of information on potential moderators of the association between screen-based SB and internalizing symptoms that may be contributing to inconsistencies in previous findings. For example, psychosocial factors associated with puberty may lead to the moderation of screen-based SB-internalizing symptom associations by sex such that screen-based SB may be related to depressive symptoms among girls, but not boys [16]. For girls in particular, puberty tends to be a time of high psychological distress, low self-esteem, and body-discontentment [17]. If consistent evidence emerges for sex as a moderator (e.g., screen-based SB-internalizing symptom associations are stronger among girls compared to boys) then heterogeneity across study findings may be attributed, in part, to moderation by sex that previous investigations may have failed to take into account. Overall, identifying the variables that strengthen or weaken the association between screen-based SB and internalizing symptoms is essential to identifying sources of inconsistencies in the existing literature, vulnerable populations, and directions for future research dedicated to optimizing intervention strategies.

Taken together, the goal of this review was to address this literature gap by providing a comprehensive overview and systematically integrate the results of observational studies assessing moderators of screen-based SB-depressive and anxiety symptom associations. Thus, the aims of this paper were to 1) summarize the moderating variables of the association between self-reported screen-based SB (e.g., television viewing, computer use) and depression and anxiety (or depressive and anxiety symptoms) among clinical and nonclinical samples of youth, 2) discuss potential mechanisms of moderation based on the consistency of evidence for particular moderators, and 3) pose suggestions for future research aimed at informing tailored intervention strategies among vulnerable populations.

## Methods

The review was performed in accordance with the Preferred Reporting Items for Systematic Reviews and Meta-Analyses (PRISMA) guidelines [18].

### Search strategy

Until December 2018, JZ and KI separately queried the Medline/PubMed, psycINFO, and Scopus electronic databases using the following search terms: (sedentary OR sitting OR screen time OR media) AND (mental health OR anxiety OR anxious OR depress* OR emotional OR internalizing OR social phobia OR panic disorder) AND (child* OR adolescents OR adolescence OR youth). Titles and abstracts were first evaluated. For those identified as possibly relevant, full texts were retrieved and assessed for inclusion in this review. Reference lists of relevant review articles were also examined.

### Study selection criteria

Peer-reviewed articles reporting on human studies published in English were included. Studies were included if a screen-based SB-anxiety symptom or screen-based SB-depressive symptom association was estimated (or if group differences were tested), and if moderation was investigated by statistically testing interaction terms or by stratifying analyses (e.g., by sex) without a formal statistical test for interaction. In addition, studies that reported estimates of associations in a common sample separately across different variants of a construct (e.g., screen-based SB – stratifying analyses by TV viewing, computer use, and videogame playing) were also considered for evidence of moderation. Studies that reported on composite variables comprised of both, screen-based and non-screen-based SB were not included in the current review because screen-based SB-specific associations could not be parsed out. Further, although they may provide specific mechanistic explanations for screen-based SB-internalizing symptom associations, studies that reported on variants within the screen-based SB construct beyond screen-type, such as specific content, were considered outside of the scope of this review. Studies utilizing any measure of anxiety or depressive symptoms, not limited to a clinical diagnosis, and including screening questions were eligible. Disagreement between JZ and KI regarding articles to be included in the review was addressed with discussion until a consensus was met.

### Data extraction and evidence synthesis

Pertinent information from each of the identified full-text studies was extracted, including authors, year of publication, sample parameters (sample size and mean age), study design, measures of screen-based SB and internalizing symptoms, moderators tested, and study results (parameter estimates) which also included significance of these moderators (amplifier/buffer/null). An amplifier was a stratum of a variable that strengthened the association between screen-based SB and internalizing symptoms, while buffers weakened the strength of the observed relationships. On occasions when a moderator resulted in a significantly *protective* association between screen-based SB and internalizing symptoms among a particular group and a null association among another group, it was considered as evidence of a buffering effect. For example, if screen-based SB were protective against internalizing symptoms among girls and unrelated to internalizing symptoms among boys, then female sex was considered a buffer. Strata of the same variable that showed comparable associations between screen-based SB and internalizing symptoms were considered null. For example, if analyses were stratified by sex, and both sedentary boys and girls experienced greater odds of depressive symptoms (with highly overlapping confidence intervals), then sex as a moderator was considered null. Percent difference in effect estimates between strata and sample size were also considered when determining the strength of the evidence for moderation on occasions where overlapping confidence intervals were present or when confidence intervals were not provided.

Consistent with previous systematic reviews [19, 20], if 0–33% of studies of a particular moderating variable provided evidence of significant moderation, then the summary result was classified as null; if 34–59% of studies of a particular moderating variable provided evidence for significant moderation, then the summary result classified as inconsistent. An inconsistent summary result also occurred in the event that fewer than four individual studies tested a particular variable as a moderator. Lastly, if 60% or more of studies of a moderating variable provided evidence of significant moderation, then the summary result was classified as significant.

### Methodological quality assessment

JZ and KI independently rated the methodological quality of each study using a modified version of an eight-component rating scale [21], consistent with a previous systematic review of SB and risk for anxiety [22]. Methodological quality was scored based on six components of the abovementioned tool: (1) selection bias (representativeness of the sample), (2) study design (cross-sectional vs. longitudinal), (3) confounders (controlling for demographic characteristics and body mass index), (4) data collection tools (valid and reliable), (5) withdrawals and dropouts (percentage of participants providing full data), and (6) appropriateness of analyses for the study design. The two components from the original were not relevant to observational studies and were therefore not included in our quality assessment were (1) blinding component and (2) other intervention-specific criteria. Each of the six above components were individually rated as weak, moderate, or strong. If a component was not described in enough detail to assign a rating, it was rated as weak. Once all six components of a study were rated, the study was given an overall rating. Studies were rated as (1) weak, if two or more individual components were rated as weak, (2) moderate, if less than three components were rated as strong with no more than one component with a weak rating, or (3) strong, if three or more study components were rated as strong.

## Results

### Overview of the studies

**Fig. 1:**
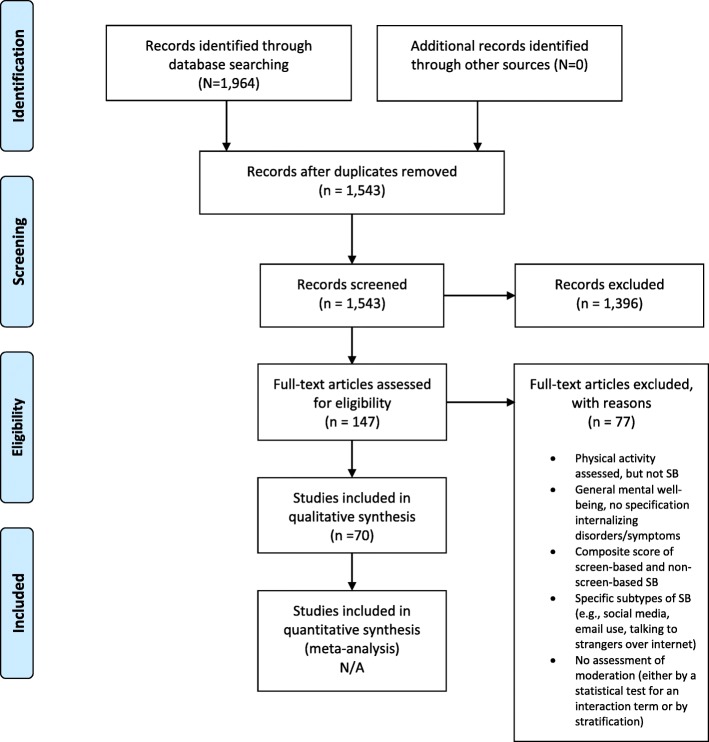
PRISMA 2009 Flow Diagram of Study Selection

Figure 1 presents the PRISMA flow of study selection. The initial literature search yielded 1964 articles. After removal of duplicates, 1543 articles remained. After screening titles and abstracts for relevance, 147 full-text articles were retrieved for further review. Seventy full-text articles tested moderation and were included. Forty-six of these studies were cross-sectional, while 19 were longitudinal, and five were both. Two of these articles assessed anxiety symptoms only, 40 assessed depressive symptoms only, and 28 investigated both internalizing symptoms and their relationship with screen-based SB. Further, of the 70 studies included in this review, only four were among those with clinical diagnoses of internalizing disorders (as determined by a physician) across three unique samples. Additional characteristics of these studies, including methodological quality score, are presented in Table 1. Interrater reliability (Cohen’s Kappa) between JZ and KI regarding manuscripts to be included in the review was 0.77, indicating substantial agreement between raters [91]; there was 80% agreement on methodological quality scores between raters. The remainder of this section is organized by the moderators that were assessed within the original studies.

**Table 1:** Characteristics of the studies included in this review (*N* = 70)

Name, Year, Reference	N (mean age, years)	Study Design	Measure of Sedentary Behavior	Measure of Depression and/or Anxiety Symptoms	Results	Methodolo-gical Quality Score
Allen, 2015 [23]	7818 (ages 6–10 at baseline)	C/L (screen-based SB predicting internalizing symptoms)	Parent-reported average mins/day of TV viewing and videogame playing on weekdays and weekend days	Depression and Anxiety: SDQ, emotional subscale	**Female sex, null (cross-sec. and long.)** Screen-based SB *sex interaction, NS, not reported **Parental education, null (cross-sec. and long.)** Screen-based SB*parental education interaction, NS, not reported **General health rating, null (cross-sec. and long.)** Screen-based SB* general health rating interaction, NS, not reported **Pubertal status, null (cross-sec. and long.)** Screen-based SB* pubertal status interaction, NS, not reported **Household income, null (cross-sectional and longitudinal)** Screen-based SB* household income interaction, NS, not reported **Neighborhood socioeconomic status, null (cross-sectional and longitudinal)** Screen-based SB* neighborhood socioeconomic status interaction, NS, not reported	Moderate
Baer, 2012 [24]	109 (13.8)	C	Self-reported hours/day of TV viewing, recreational computer use, and videogame playing	Depression and Anxiety: SDQ, emotional subscale	**TV viewing, null** (Standardized regression coefficients presented) TV viewing:0.08 ± 0.09 (*p* > .05) Computer use:0.05 ± 0.11 (*p* > .05) Videogames: −0.13 ± 0.10 (*p* > .05)	Weak
Barcaccia, 2018 [25]	301 (17.1)	C	Self-reported average daily time spent watching TV	Depression: Beck Depression Inventory (BDI) Anxiety: State-Trait Anxiety Inventory (STAI) Form Y	**Female sex, null (dep.)** In boys: β = 0.127 (*p* > .05) In girls: β = 0.384 (*p* > .05) **Female sex, null (anx.)** In boys: β = 0.072 (*p* > .05) In girls: β = −0.046 (*p* > .05)	Weak
Bélair, 2018 [26]	9702 (ages 14–15)	C	Self-reported average daily time spent TV viewing, videogame playing, or watching videos	Depression and Anxiety: Ontario Child Health Study (7 items)	**Female sex, null (dep. and anx.)** Screen-based SB in entire sample: OR 1.36 (95%CI 1.11–1.67) Stratified analyses by sex NS different than estimate in entire sample, not reported **Physical activity, null (dep. and anx.)** Screen-based SB*physical activity interaction NS, not reported	Weak
Bélanger, 2011 [27]	7211 (ages 16–20)	C	Self-reported hours/day and days/week of internet use	Depression: Depressive Tendencies Scale	**Female sex, null** Heavy vs. regular Internet users in boys: RRR = 1.36 (95%CI 1.01–1.81) Heavy vs. regular Internet users in girls: RRR = 1.86 (95%CI 1.30–2.66)	Weak
Bickham, 2015 [28]	126 (14.0)	C/L (screen-based SB predicting depressive symptoms)	Self-reported average amount of TV viewing, computer use, videogame playing, and mobile phone use on weekends and weekdays	Depression: BDI	**TV viewing, null (cross-sectional)** TV: β = −0.07 (p = .45) Computer: β = 0.03 (*p* = .76) Videogames: β = − 0.08 (*p* = 0.39) Mobile phone: β = − 0.04 (*p* = .66) **TV viewing and mobile phone use, amplifier (longitudinal)** TV: β = 0.21 (*p* = .03) Computer: β = 0.08 (*p* = .45) Videogames: β = 0.004 (*p* = .97) Mobile phone: β = 0.22 (*p* = .02)	Moderate
Brodersen, 2005 [29]	4320 (11.8)	C	Self-reported average TV viewing, computer use, and videogame playing on weekends and weekdays	Depression and Anxiety: SDQ, emotional subscale	**Female sex, amplifier** In boys: β = 0.08 (*p* = .48) In girls: β = 0.34 (*p* = .001)	Weak
Cao, 2011 [30]	5003 (13.2)	C	Self-reported average hours of TV viewing and computer use outside of school hours	Depression: Depression Self-Rating Scale for Children (DSRSC) Anxiety: Screen for Child Anxiety Related Emotional Disorders (SCARED)	**Vigorous physical activity (VPA), buffer (dep.)** High screen-based SB and low VPA: REF. Low screen-based SB and low VPA: OR 0.66 (95%CI 0.56–0.79) High screen-based SB and high VPA: OR 0.78 (95%CI 0.60–1.01) Low screen-based SB and low VPA: OR 0.50 (0.41–0.62) **VPA, buffer (anx.)** High screen-based SB and low VPA: REF. Low screen-based SB and low VPA: OR 0.72 (95%CI 0.60–0.85) High screen-based SB and high VPA: OR 0.87 (95%CI 0.67–1.12) Low screen-based SB and low VPA: OR 0.67 (0.41–0.62)	Strong
Casiano, 2012 [31]	9137 (ages 12–19)	C	Self-reported average hours per week of TV viewing, computer use, and videogame playing	Depression: Composite International Diagnostic Interview (CIDI)	**Videogame use, buffer** TV: OR 0.94 (95%CI 0.87–1.02) Computer: OR 1.03 (95%CI 0.96–1.10) Videogames: OR 0.87 (95%CI 0.79–0.97)	Weak
Castillo, 2014 [32]	1508 (13.9)	C	Self-reported time spent in screen-based SB on the most recent non-school day (TV viewing, computer use, videogames, listening to music, and telephone/texting)	Depression: Kandel’s 6-item depressive symptoms scale	**Female sex, null** Depression*sex interaction (*p* = .07)	Weak
Chen, 2009 [33]	10,347 (grade 11)	L (screen-based SB predicting depressive symptoms)	Self-reported average time watching TV and playing Internet games after school	Depression: Center for Epidemiological Studies Depression Scale (CES-D) and BDI	**Internet games, buffer** TV: β = 0.00, *p* > 0.05 Internet games: β = −0.31, *p* < .01	Strong
Cho, 2017 [34]	10,261 (ages 11–17 at baseline)	L (depressive symptoms predicting screen-based SB)	Self-reported weekly hours of TV viewing, video viewing, computer games, and videogames.	Depression: Modified CES-D	**Socioeconomic Neighborhood Environment, null** Depressive symptom*poverty interaction NS, not reported Depressive symptom*education interaction NS, not reported Depressive symptom*Race/ethnicity composition interaction NS, not reported	Weak
Dennison-Farris, 2017 [35]	121 (10.5)	C	Self-reported average daily hours of TV viewing, computer use, videogame playing, and tablet use on weekdays and weekend days	Depression: Children’s Depression Inventory (CDI)	**Tablet, amplifier (on weekdays only)** *On weekdays* TV: β = 0.01 (*p* = .65) Computer: β = 0.03 (*p* = .06) Videogames: β = 0.01 (*p* = .66) Tablet: β = 0.03 (*p* = .03) **Tablet, null (on weekend days only)** *On weekend days* TV: β = 0.04 (*p* = .01) Computer: β = 0.04 (*p* = .02) Videogames: β = 0.03 (*p* = .03) Tablet: β = 0.03 (*p* = .04)	Weak
Desai, 2010 [36]	4028 (ages 14–18)	C	Self-reported average time playing video or computer games in the previous week	Depression: self-reports of being sad or hopeless for 2 weeks or more during the previous year	**Female sex, buffer** Depressive symptom*sex interaction (*p* = .01) In boys: OR 1.15 (*p* = .35) In girls: OR 0.72 (*p* = .002)	Weak
Do, 2013 [37]	136,589 (ages 13–18)	C	Self-reported average daily minutes of Internet use (non-study purposes) over the last 30 days	Depression: self-reports of sadness/despair or suicidal ideation	**Longer sleep duration, null** Internet*sleep duration interaction NS, not reported	Weak
Durkin, 2002 [38]	1304 (16.0)	C	Self-reported of average computer game use	Depression: 4-item scale (measure not reported)	**Female sex, null** Screen-based SB*sex interaction NS, not reported	Weak
Fulkerson, 2004 [39]	4734 (14.8)	C	Self-reported average TV viewing on weekends and weekdays	Depression: Kandel and Davies 6-item depressive mood scale	**Female sex, amplifier (on weekdays and weekend days)** *On weekdays* Mean hours of screen-based SB in boys: Low dep = 4.7 h vs. moderate dep. = 4.8 h vs high dep. = 4.8 h (*p* = .665) Mean hours of screen-based SB in girls: Low dep. = 4.3 h vs. moderate dep. = 4.7 h vs. high dep. = 4.5 h (*p* < .001) *On weekend days* Mean hours of screen-based SB in boys: Low dep = 4.7 h vs. moderate dep. = 4.7 h vs. high dep. = 4.7 h (*p* = .705) Mean hours of screen-based SB in girls: Low dep. = 4.3 h vs. moderate dep. = 4.6 h vs. high dep. = 4.3 h (*p* < .001)	Weak
Godinho, 2014 [40]	1680 (aged 13)	C	Self-reported average TV viewing and computer use on weekends and weekdays	Depression: BDI-II	**Female sex and computer use, amplifier** TV (in boys): OR 1.17 (95%CI 0.64–2.14) TV (in girls): NS, not reported Computer (in boys): OR 1.02 (95%CI 0.55–1.89) Computer (in girls): OR 1.71 (95%CI 1.11–2.61)	Moderate
Goldfield, 2016 [41]	358 (15.6)	C	Self-reported average daily hours TV viewing, computer use, and playing inactive videogames	Depression: CDI	**Female sex, null** Total screen-based SB*sex interaction (*p* = .751) TV*sex interaction (*p* = .276) Computer*sex interaction (*p* = .623) Videogame*sex interaction (*p* = .335) **TV Viewing, buffer** TV: β = 0.11 (*p* > .05) Computer: β = 0.18 (*p* = .006) Videogames: β = 0.13 (p = .05)	Weak
Gopinath, 2012 [42]	1094 (17.3) (C) 775 (17.3) (L)	C/L (screen-based SB predicting internalizing symptoms)	Self-reported average daily hours TV viewing, computer use, videogame playing	Depression and Anxiety: Pediatric Quality of Life Inventory (PedsQL), emotional subscale	**TV viewing, null (dep. and anx.) (cross-sectional)** TV Viewing: β = −2.4, *p* = .01 Computer: β = − 2.3, *p* = .004 Videogames: β = −3.2, *p* = .01 **TV viewing, buffer (dep. and anx.)** **(longitudinal)** Low TV viewing mean score = 76.17 Moderate TV Viewing mean score = 73.78 High TV viewing mean score = 71.31 (p-trend = .10) Low total screen time mean score = 75.31 Moderate total screen time mean score = 76.92 High total screen time mean score = 66.98 (p-trend = .01)	Moderate
Griffiths, 2010 [43]	13,470 (5.2)	C	Mother-report of child’s average hours of TV/videos/DVD, computer use, and electronic game use	Depression and Anxiety: SDQ, emotional subscale	**Female sex, amplifier (dep. and anx.)** In boys: β = − 0.03, *p* = .77 In girls: β = 0.09, *p* = .03	Moderate
Grontved, 2015 [44]	435 (aged 15 at baseline)	L (screen-based SB predicting depressive symptoms)	Self-reported leisure time TV viewing and computer use	Depression: Major Depression Inventory (MDI)	**Female sex, null** TV*sex interaction (*p* = .89) Computer*sex interaction (*p* = .18) **TV Viewing, amplifier** TV: β = 1.24 (*p* < .001) Computer: β = −.06 (*p* = .91)	Weak
Hamer, 2016 [45]	2038 (16.0)	L (screen-based SB predicting internalizing symptoms)	Self-reported time spent TV viewing, watching videos, and computer use “after school yesterday”	Depression and Anxiety: 9-item Malaise Inventory	**Female sex, null (dep. and anx.)** Screen-based SB*sex interaction NS, not reported	Moderate
Hayward, 2016 [46]	3295 (15.1)	C	Self-reported screen-based SB from single item on Core Indicators and Measures of Youth Health Survey	Depression: Short Mood and Feelings Questionnaire (MFQ)	**Female sex, buffer** In boys: OR 0.98 (95%CI 0.93–1.03) In girls: OR 0.95 (95%CI 0.91–0.98)	Moderate
Hinkley, 2014 [47]	3604 (4.3)	L (screen-based SB predicting internalizing symptoms)	Parent-reported average daily hours of TV viewing and e-game/computer use on weekdays and weekend days	Depression and Anxiety: SDQ, emotional subscale	**Female sex and TV viewing, null (dep. and anx.) (on weekdays and weekend days)** *On weekdays* TV viewing in boys: OR 1.2 (95% CI 0.9–1.5) TV viewing in girls: OR 1.3 (95%CI 1.0–1.7) Computer use in boys: OR 1.3 (95%CI 0.8–2.1) Computer use in girls: OR 2.0 (95%CI 1.0–4.0) *On weekend days* TV viewing in boys: OR 1.0 (95% CI 0.8–1.3) TV viewing in girls: OR 1.3 (95% CI 1.0–1.6) Computer use in boys: OR 1.0 (95%CI 0.7–1.4) Computer use in girls: OR 1.1 (95%CI 0.7–1.8)	Moderate
Hoare, 2014 [48]	800 (13.1)	C	Self-reported average daily hours of TV viewing, computer use, and videogame playing	Depression: SMFQ	**Female sex, null** In boys: OR 1.22 (*p* = .01) In girls: OR 1.12 (*p* = .02)	Weak
Holtz, 2011 [49]	205 (12.7)	C	Self-reported average daily hours of Internet use (for communication, informational purposes, and online gaming)	Depression and Anxiety: German version of the Youth Self Report (YSR), internalizing subscale	**Parental communication, null (dep. and anx.)** Parent communication*Internet use interaction NS (Wald χ^2^ < 0.6)	Weak
Houghton, 2018 [50]	1749 (ages 10–17)	L (bi-directional)	Self-reported screen-based SB (TV viewing, web, gaming, and social media) via Screen Based Media Use Scale	Depression: CDI 2	**Female sex, buffer** **Social media, amplifier** TV viewing in boys: β = 0.108 (*p* = .058) (depressive symptoms predicting TV viewing) β = 0.057 (*p* = .290) (TV viewing predicting depressive symptoms) Web use in boys: β = 0.058 (*p* = .246) (depressive symptoms predicting web use) β = 0.115 (*p* = .028) (web use predicting depressive symptoms) Gaming in boys: β = 0.045 (*p* = .338) (depressive symptoms predicting gaming) β = 0.040 (*p* = .451) (gaming predicting depressive symptoms) Social media in boys: β = 0.163 (*p* = .002) (depressive symptoms predicting social media) β = 0.150 (*p* = .002) (social media predicting depressive symptoms) TV viewing in girls: β = 0.033 (*p* = .563) (depressive symptoms predicting TV viewing) β = 0.072 (*p* = .212) (TV viewing predicting depressive symptoms) Web use in girls: β = − 0.022 (*p* = .707) (depressive symptoms predicting web use) β = 0.070 (*p* = .211) (web use predicting depressive symptoms) Gaming in girls: β = 0.038 (*p* = .404) (depressive symptoms predicting gaming) β = 0.050 (*p* = .302) (gaming predicting depressive symptoms) Social media in girls: β = − 0.061 (*p* = .238) (depressive symptoms social media) β = − 0.083 (*p* = .104) (social media predicting depressive symptoms) **Mid-adolescence, amplifier** In boys (early): β = 0.032 (*p* = .757) (depressive symptoms predicting total screen-based SB) β = 0.361 (*p* = .241) (total screen-based SB predicting depressive symptoms) In girls (early): β = − 0.054 (*p* = .555) (depressive symptoms predicting total screen-based SB) β = 0.002 (*p* = .986) (total screen-based SB predicting depressive symptoms) In boys (mid): β = 0.068 (*p* = .501) (depressive symptoms predicting total screen-based SB) β = 0.272 (*p* < .001) (total screen-based SB predicting depressive symptoms) In girls (mid): β = 0.217 (*p* = .107) (depressive symptoms predicting total screen-based SB) β = 0.253 (*p* = .024) (total screen-based SB predicting depressive symptoms) In boys (late): β = − 0.009 (*p* = .955) (depressive symptoms predicting total screen-based SB) β = 0.107 (*p* = .518) (total screen-based SB predicting depressive symptoms) In girls (late): β = 0.026 (*p* = .857) (depressive symptoms predicting total screen-based SB) β = 0.187 (*p* = .302) (total screen-based SB predicting depressive symptoms)	Moderate
Hrafnkelsdottir, 2018 [51]	244 (15.8)	C	Self-reported average daily hours of TV viewing, computer use, and videogame playing on weekends and weekdays	Depression and Anxiety: Symptom Checklist 90 (SCL-90) depression and anxiety subscales	**VPA, buffer (dep.)** Low VPA and high screen-based SB: REF. High VPA and low screen-based SB: RR = 0.06 (95%CI 0.01–0.41) **VPA, buffer (anx.)** Low VPA and high screen-based SB: REF. High VPA and low screen-based SB: RR = 0.16 (95%CI 0.06–0.45)	Weak
Hume, 2011 [52]	155 (14.4)	C/L (bi-directional)	Self-reported average TV viewing	Depression: CES-D	**Female sex, null (cross-sectional)** In boys: NS, not reported In girls: NS, not reported **Female sex, null (long., screen-based SB predicting depressive symptoms)** In boys: NS, not reported In girls: NS, not reported **Female sex, amplifier (long., depressive symptoms predicting screen-based SB)** In boys: β = −4.29 (*p* > .05) In girls: β = 3.53 (*p* < .001)	Moderate
Iannotti, 2013 [53]	9058 (13.9)	C	Self-reported average daily hours of TV viewing, computer use, and videogame playing on weekends and weekdays	Depression: Scale not reported, 6 screening questions (sadness, irritability, hopelessness, change in appetite, change in sleeping, and problems concentrating)	**Female sex, null** Healthful boys (low screen-based SB): mean depression score = 2.11 Unhealthful boys (high screen-based SB): mean depression score = 2.35 Typical boys (moderate screen-based SB): mean depression score = 2.17 (each group sig. Different from one another at *p* < .05 level) Healthful girls (low screen-based SB): mean depression score = 2.40 Unhealthful girls (high screen-based SB): mean depression score = 2.76 Typical girls (moderate screen-based SB): mean depression score = 2.64 (each group sig. Different from one another at *p* < .05 level)	Strong
Katon, 2010 [54]	2274 (13–17)	C	Self-reported average time TV viewing and computer use	Depression: Patient Health Questionnaire (PHQ-2)	**Computer use, amplifier** Mean hours of TV viewing: Low dep. = 1.7 vs. high dep. = 1.7 (*p* > .05) Mean hours of computer use: Low dep. = 1.6 vs. high dep. = 1.9 (*p* < .01)	Weak
Khan, 2017 [55]	505 (14.3)	C	Self-reported average daily TV viewing, DVD viewing, computer use, and social media on weekdays and weekend days	Depression: CES-D 10	**MVPA, buffer** In sedentary participants meeting the MVPA guidelines: REF. In sedentary participants not meeting the MVPA guidelines: OR 2.37 (95%CI 1.23–4.59)	Weak
Kim, 2012 [56]	75,066 (grades 7–12)	C	Self-reported internet use for non-educational purposes	Depression: depression rate via the Korea Youth Risk Behavior Web-based Survey	**Female sex, amplifier** No internet users in boys: OR 1.21 (95%CI 1.13–1.30) Occasional internet users in boys: REF. Moderate internet users in boys: OR 1.11 (95%CI 1.02–1.21) Heavy internet users in boys: OR 1.33 (95%CI 1.12–1.59) No internet users in girls: OR 1.15 (95%CI 1.06–1.25) Occasional internet users in girls: REF. Moderate internet users in girls: OR 1.33 (95%CI 1.20–1.47) Heavy internet users in girls: OR 1.87 (95%CI 1.43–2.46)	Weak
Kim, 2016 [57]	2198 (middle schoolers)	L (screen-based SB predicting depressive symptoms)	Self-reported daily average hours of videogame playing	Depression: CESD-R	**High neighborhood divorce rate, buffer** Screen-based SB*divorce interaction β = −0.171 (*p* < .05) **Neighborhood population size, null** Screen-based SB*population size interaction β = 0.003 (*p* > .05) **Neighborhood education level, null** Screen-based SB*education interaction β = − 0.006 (*p* > .05)	Strong
Kleppang, 2019 [58]	3223 (ages 15–16)	C	Self-reported average daily hours of TV viewing, computer use, and videogame playing	Depression and Anxiety: Hopkins Symptom Checklist-10 (HSCL-10)	**Female sex, null** Sex*screen-based SB interaction NS, not reported **Year of investigation, null** Year*screen-based SB interaction NS, not reported	Moderate
Krejci, 2011 [59]	599 (3.8) (Japanese) 497 (4.6) (Czech)	C	Parent-reported average use of videogames	Depression: Morningness-eveningness questionnaire (MEQ) and original sleep habit questionnaire	**Japanese origin, buffer** In Japanese children: χ^2^-value = 14.17 (p = .028) In Czech children: χ^2^-value = 5.83 (*p* = 0.757)	Weak
Kremer, 2013 [60]	8029 (11.5)	C	Self-reported screen-based SB via the Communities That Care Youth Survey (TV viewing, computer use, and videogame playing)	Depression: Short MFQ	**Female sex, null** Screen-based SB*sex interaction *p* = .91 **Younger age, amplifier** Screen-based SB*age interaction p = .04 In older participants: Conditional OR 0.64 In younger participants: Conditional OR 0.88 **Physical activity (PA), null** Screen-based SB*PA interaction *p* = .92	Moderate
Lemola, 2015 [61]	362 (14.8)	C	Self-reported average use of TV viewing, videogame playing, phone use/texting, and Internet use before going to sleep	Depression: six items from the CES-D (German version)	**Age, null** SB*age interaction *p* > .10 **TV viewing and phone/texting, buffer** TV: NS, not reported Videogames: β = 0.15, *p* = .005 Phone/texting: NS, not reported Internet use: β = 0.19, *p* < .001	Weak
Liu, 2016 [62]	13,369 (15.2)	C	Self-reported TV viewing and computer/videogame use via the Youth Risk Behavior Survey (YRBS)	Depression: CES-D Anxiety: Multidimensional Anxiety Scale for Children (MASC)	**Female sex and computer/videogames, amplifier (dep.)** TV in boys: OR 1.33 (95%CI 1.02–1.73) TV in girls: OR 1.62 (95% CI 1.19–2.21) Computer/videogames in boys: OR 1.61 (95%CI 1.28–2.03) Computer/videogames in girls: OR 1.96 (95%CI 1.42–2.71) **Female sex, null** **Computer/videogames, amplifier (anx.)** TV in boys: OR 1.43 (95%CI 1.05–1.95) TV in girls: OR 1.51 (95%CI 0.96–2.38) Computer/videogames in boys: OR 1.40 (95%CI 1.06–1.86) Computer/videogames in girls: OR 1.78 (95%CI 1.09–2.89)	Weak
Maras, 2015 [63]	2482 (14.1)	C	The Leisure-Time Sedentary Activities Questionnaire (TV viewing, computer use, and videogame playing)	Depression: CDI Anxiety: MASC-10	**TV viewing, buffer (dep.)** TV: β = 0.03, *p* > .05 Computer: β = 0.17, *p* < .001 Videogames: β = 0.13, *p* < .001 **Videogames, amplifier (anx.)** TV: β = 0.03, *p* > .05 Computer: β = −0.03, *p* > .05 Videogames: β = 0.11, *p* < .001	Weak
Mathers, 2009 [64]	925 (16.1)	C	Multimedia Activity Recall for Children and Adolescents (MARCA) (TV viewing, computer use, videogame playing, and telephone/texting)	Depression and Anxiety: Kessler-10	**Videogame playing, amplifier (dep. and anx.)** **Computer use, buffer (dep. and anx.)** TV: OR 1.03 (95%CI 0.62–1.70) Computer: OR 0.61 (95%CI 0.38–0.96) Videogames: OR 1.79 (95%CI 1.17–2.73) Telephone/texting: OR 1.13 (95%CI 0.74–1.74)	Weak
McHale, 2009 [65]	469 youth (12.8 & 15.7)	C	Self-reported TV viewing	Depression: CES-D	**Low parental education values, amplifier** TV in those with parents with high education values: NS, not stated TV in those with parents with low education values: λ = −.002 (*p* < .05) **Parental cultural orientations, null** TV viewing*cultural orientations interaction NS, not reported	Weak
McVeigh, 2016 [4]	2411 (aged 1 at baseline)	L (screen-based SB predicting internalizing symptoms)	Parent-reported and self-reported TV viewing	Depression and Anxiety: Depression Anxiety Stress Scales (DASS-21)	**Female sex, null (dep. and anx.)** In boys: RR 1.00 (95%CI 0.72–1.38) (dep. and anx.) In girls: RR 1.09 (95%CI 0.87–1.36) (dep. and anx.)	Moderate
Mistry, 2007 [66]	2707 (ages 30–33 months at baseline)	L (screen-based SB predicting internalizing symptoms)	Parent-reported TV viewing	Depression and Anxiety: Child Behavior Checklist (CBCL) internalizing subscale	**Household income, null (dep. and anx.)** Screen-based SB*household income interaction NS, not reported **Parental involvement, null (dep. and anx.)** Screen-based SB*parental involvement interaction NS, not reported	Moderate
Mundy, 2017 [67]	876 (9.0)	C	Parent-reported average weekly time spent in TV viewing, computer use, and videogame playing	Depression and Anxiety: SDQ, emotional subscale	**Videogame playing, amplifier (dep. and anx.)** **Female sex, buffer (dep. and anx.)** TV in boys: OR 1.00 (95%CI 0.96–1.03) TV in girls: OR 1.02 (95%CI 0.99–1.05) Computer in boys: OR 0.98 (95%CI 0.94–1.02) Computer in girls: OR 1.04 (95%CI 0.99–1.08) Videogames in boys: 1.07 (95%CI 1.04–1.11) Videogames in girls: 1.03 (95%CI 0.98–1.08)	Weak
Nakamura, 2012 [68]	3464 (10.1)	C	Self-reported average daily TV viewing, computer use, and videogame playing	Depression: single-item on subjective health complaint scale	**TV Viewing, buffer** TV: NS, not reported Computer: effect estimate not reported-positive association (*p* < .001) Videogames: effect estimate not reported-positive association, (*p* = .001)	Weak
Ohannessian, 2009 [69]	328 (15.0)	C/L (screen-based SB predicting internalizing symptoms)	Self-reported average daily TV viewing, emailing/IM-ing/surfing the web, videogame playing, and text messaging	Depression: CES-DC Anxiety: SCARED	**Female sex, amplifier (dep.)** **Surfing the web, null (dep.)** Screen-based SB*sex interaction (*p* < .05) (TV viewing) TV: NS, not reported Surfing the web: NS, not reported (cross-sec. and long.) Videogames: NS, not reported (cross-sec. and long.) Texting: NS, not reported (cross-sec. and long.) **Parental alcoholism, null (dep.)** Parental alcoholism* screen-based SB interaction NS, not reported **Female sex, amplifier (anx.)** Screen-based SB*sex interaction (*p* < .05) (TV viewing and videogames) **Surfing the web, amplifier (anx.)** TV: NS, not reported (cross-sec. and long.) Surfing the web: F (7,154) = 6.02 (*p* < .05) (long. only) Videogames: NS, not reported (cross-sec. and long.) Texting: NS, not reported (cross-sec. and long.) **Parental alcoholism, amplifier (anx.)** Parental alcoholism*surfing the web interaction (*p* < .05) (cross-sec. and long.) Parental alcoholism*sex*videogame interaction (*p* < .05) (cross-sec.)	Weak
Ohannessian, 2018 [70]	441 (17.1)	L (screen-based SB predicting anxiety symptoms)	Self-reported average daily videogame playing	Anxiety: SCARED	**Female sex and playing with others (social context), amplifiers** Videogame*sex interaction *p* < .001 Videogame*sex*social context interaction *p* < .01	Weak
Parkes, 2013 [71]	11,014 (age 5 at baseline)	L (screen-based SB predicting internalizing symptoms)	Parent-reported average daily hours of TV viewing and computer/videogame playing	Depression and Anxiety: SDQ, emotional subscale	**Female sex, null (dep. and anx.)** Screen-based SB*sex interaction *p* > .05 (TV viewing and computer/videogames) **TV Viewing, null (dep. and anx.)** TV 0 h daily: β = −0.05 (*p* = .66) TV < 1 h daily: REF. TV 1–3 h daily: β = 0.02 (*p* = .64) TV > 3 h daily: β = 0.01 (*p* = .91) Computer/videogames 0 h daily: β = 0.07 (*p* = .09) Computer/videogames < 1 h daily: REF. Computer/ videogames 1–3 h daily: β = 0.03 (*p* = .48) Computer/videogames > 3 h daily: β = 0.26 (*p* = .11)	Moderate
Poulain, 2018 [72]	527 (3.8)	L (bi-directional)	Parent-reported average hours of TV viewing, videogames, computer use, and mobile phone use	Depression and Anxiety: SDQ, emotional subscale	**Female sex, null (dep. and anx., bi-directionally)** Screen-based SB*sex interaction *p* > .05 **Age, null (dep. and anx., bi-directionally)** Screen-based SB*age interaction *p* > .05 **Socioeconomic status, null (dep. and anx., bi-directionally)** Screen-based SB*socioeconomic status interaction *p* > .05 **TV viewing, null (dep. and anx., bi-directionally)** *Screen-based SB predicting internalizing symptoms* TV: OR 0.63 (95%CI 0.35–1.12) Computer: OR 0.98 (95%CI 0.35–2.77) Videogames: OR 0.47 (95%CI 0.09–2.34) Mobile phone: OR 0.68 (95%CI 0.17–2.68) *Internalizing symptoms predicting screen-based SB* TV: OR 0.96 (95%CI 0.80–1.16) Computer: OR 0.96 (95%CI 0.78–1.18) Videogames: OR 1.10 (95%CI 0.87–1.39) Mobile phone: OR 0.87 (95%CI 0.66–1.15)	Moderate
Primack, 2009 [73]	4142 (21.8 at follow-up)	L (screen-based SB predicting depressive symptoms)	Self-reported average hours of TV viewing, video viewing, and videogames	Depression: CES-D	**Female sex, buffer** Screen-based SB*sex interaction OR 0.93 (95%CI 0.88–0.99) **TV viewing, amplifier** TV: OR 1.08 (95%CI 1.01–1.06) Videos: OR 1.03 (95%CI 0.86–1.25) Videogames: OR 1.04 (95%CI 0.89–1.22)	Strong
Primack, 2011 [74]	106 (12.7)	C	Self-reported media use (TV viewing, videogames, and computer use)	Depression: DSM-III or DSM-IV diagnostic criteria for major depressive disorder	**Female sex, null** Screen-based SB*sex interaction term NS, not reported **Age, null** Screen-based SB*age interaction term NS, not reported **TV viewing, null** TV: OR 1.2 (95%CI 0.8–1.7) Computer: OR 1.1 (95%CI 0.7–1.6) Videogames: OR 1.3 (95% CI 0.8–2.1)	Weak
Robinson, 2011 [75]	1860 (14.0)	C	Self-reported average daily hours of TV viewing and computer use	Depression and Anxiety: CBCL (internalizing subscale)	**Female sex, null** Screen-based SB*sex interaction NS, not reported	Weak
Romer, 2013 [76]	719 (ages 12–24)	L (screen-based SB predicting depressive symptoms)	Self-reported average time TV viewing, computer use, and videogames	Depression: Youth Risk Behavior Survey (2 items, reports of sadness or hopelessness)	**Younger age, null** Screen-based SB*age interaction NS, not reported **TV viewing, buffer** TV: NS, not reported Computer: β = 0.12 (*p* = .039) Videogames: β = 0.14 (*p* = .001)	Weak
Rottenberg, 2014 [77]	363 (15.8–17.0)	L (depression predicting screen-based SB)	Self-reported TV viewing and computer use via YRBS	Depression: Interview Schedule for Children and Adolescents Diagnostic Version (ISCA-D)	**TV viewing, null** TV: OR 1.53 (95%CI 1.19–1.95) Computer: OR 1.31 (95%CI 1.05–1.62)	Moderate
Schmitz, 2002 [78]	3798 (12.8)	L (depressive symptoms predicting screen-based SB)	Self-reported average time of TV viewing and playing videogames	Depression: CES-D	**Female sex, null** In boys: mean sed. Score amongst those with most depressive symptoms = 12.66 (95%CI 12.24–13.08) (*p* < .01) In girls: mean sed. Score amongst those with most depressive symptoms = 10.28 (95%CI 9.99–10.58) (*p* < .01)	Moderate
Schreck, 2016 [79]	178 (11.3)	C	Parent-reported average time spent TV viewing, using the computer, and playing videogames	Depression: CBCL, withdrawn/depressed subscale	**Female sex, null** In boys: β = 0.05 (*p* > .05) In girls: β = 0.05 (*p* > .05)	Weak
Selfhout, 2009 [80]	307 (15.5)	L (screen-based SB predicting internalizing symptoms)	Self-reported average time spent instant messaging or surfing the web	Depression: CDI Anxiety: SCARED	**Female sex, null (dep. and anx.)** screen-based SB*sex interaction *p* > .05 (instant messaging and surfing the web) **Low perceived friendship quality (surfing the web), amplifier (dep. and anx.)** **Low perceived friendship quality (instant messaging), buffer (dep. only)** Screen-based SB*friendship quality interaction β = − 0.23 (*p* < .01) (dep.) (instant messaging) Screen-based SB*friendship quality interaction β = 0.20 (*p* < .01) (dep.) (surfing the web) Screen-based SB*friendship quality interaction β = .03 (*p* > .05) (anx.) (instant messaging) Screen-based SB*friendship quality interaction β = .13 (*p* < .01) (anx.) (surfing the web) High instant messaging and low friendship quality: β = − 0.27 (*p* < .01) vs. high instant messaging and high friendship quality: β = 0.02 (*p* > .05) (dep.) High surfing the web and low friendship quality: β = 0.33 (*p* < .01) vs. high surfing the web and high friendship quality: β = 0.05 (*p* > .05) (dep.) High surfing the web and low friendship quality: β = 0.22 (*p* < .01) vs. high surfing the web and high friendship quality: β = 0.03 (*p* > .05) (anx.) **Female sex and low perceived friendship quality, null (dep. and anx.)** Screen-based SB*sex*friendship quality interaction *p* > .05 (instant messaging and surfing the web)	Moderate
Singer, 1998 [81]	2245 (11.0)	C	Self-reported daily average time watching TV	Depression and Anxiety: Trauma Symptom Checklist for Children (TSCC), depression and anxiety subscales	**Female sex, amplifier (dep.)** Depression rate in boys: low TV = 7.0%, high TV = 10.6% (*p* > .05) Depression rate in girls: low TV = 5.8%, high TV = 17.8% (*p* < .01) **Female sex, null (anx.)** Anxiety rate in boys: low TV = 5.5%, high TV = 12.4% (*p* < .01) Anxiety rate in girls: low TV = 7.0%, high TV = 15.3% (*p* < .01)	Weak
Straker, 2013 [82]	643 (14.0)	C	MARCA	Depression: BDI Depression and Anxiety: CBCL, internalizing subscale	**Female sex, buffer (dep., BDI)** Computer (instrumental) in boys: median (IQR) = 3.0 (4.0) vs. computer (for games): median (IQR) = 4.0 (7.0) vs. videogames: median (IQR) = 4.0 (7.0) (*p* = .046) Computer (instrumental) in girls: median (IQR) = 5.0 (8.0) vs. computer (for games): median (IQR) = 5.0 (9.0) vs. videogames: Not reported (*p* = .995) **Female sex, null (dep. and anx., CBCL)** Computer (instrumental) in boys: median (IQR) = 7.4 (5.0) vs. computer (for games): median (IQR) = 10.0 (6.8) vs. videogames: median (IQR) = 9.1 (6.1) (*p* = .071) Computer (instrumental) in girls: median (IQR) = 10.2 (6.5) vs. computer (for games): median (IQR) = 10.2 (6.6) vs. videogames: Not reported (*p* = .950)	Weak
Suchert, 2015 [16]	1296 (13.7)	C	Self-reported time spent TV viewing, computer use, videogames, and mobile phone on the most recent school day and the most recent Sunday	Depression: CES-D (depressed affect subscale)	**Female sex, amplifier** In boys: β = −0.024 (*p* = .552) In girls: β = 0.094 (*p* = .032)	Moderate
Trinh, 2015 [83]	2660 (15.8)	C	Self-reported daily average time spent TV viewing, computer use, and videogame playing	Depression: CES-D (4 items: sad, lonely, depressed, crying)	**Female sex, buffer** In boys: OR 2.82 (95%CI 1.09–7.30) In girls: OR 1.65 (95%CI 0.87–3.16) **PA, null** Screen-based SB*PA interaction NS, not reported	Weak
Twenge, 2018 [84]	388,275 (grades 8–10)	C	Self-reported average daily time spent TV viewing, social media use, and internet use for news	Depression: 6 items from the Bentler Medical and Psychological Functioning Inventory, depression subscale	**Social Media and Female sex, amplifier** TV in boys: r = 0.02 (*p* < .05) Internet for news in boys: r = −0.02 (*p* > .05) Social media in boys: r = 0.01 (*p* > .05) TV in girls: r = 0.03 (*p* < .001) Internet for news in girls: r = 0.01 (*p* > .05) Social media in girls: r = 0.06 (*p* < .001) **Low in-person social interaction, amplifier** Social media*social interaction *p* < .001 Social media among those with low in-person social interaction: *F*(1, 9765) = 185.35 (*p* < .001) Social media among those with high in-person social interaction: *F*(1, 11,271) = 2.21 (*p* = .14)	Weak
Whiteley, 2011 [85]	1518 (15.3)	C	Self-reported average weekly computer use and mobile phone use	Depression: CES-D8	**Computer use, null** Computer: β = 0.01 (*p* = .777) Mobile phone: β = 0.001 (*p* = .980)	Weak
Wu, 2017 [86]	4875 (ages 10–11)	L (screen-based SB predicting depression and anxiety)	Self-reported average daily time spent TV viewing, computer use, and videogame playing	Depression and Anxiety: Clinical diagnoses defined by the International Classification of Diseases, Ninth Revision, Clinical Modification or Tenth Revision	**TV viewing, buffer (dep. and anx.)** TV < 1 h/day, REF. TV 1–2 h/day, IRR 0.98 (95CI 0.74–1.29) TV 3–4 h/day, IRR 0.93 (95%CI 0.71–1.23) TV 5h hours/day, IRR 0.77 (95%CI 0.56–1.06) Computer/videogames < 1 h/day, REF. Computer/videogames 1–2 h/day, IRR 1.56 (95%CI 1.26–1.93) Computer/videogames 3–4 h/day, IRR 1.42 (95%CI 1.06–1.91) Computer/videogames 5h hours/day, IRR 1.67 (95%CI 1.10–2.54)	Moderate
Wu, 2018 [87]	4861 (ages 10–11)	L (screen-based SB predicting depression and anxiety)	Self-reported average daily time spent TV viewing, computer use, and videogame playing	Depression and Anxiety: Clinical diagnoses defined by the International Classification of Diseases, Ninth Revision, Clinical Modification or Tenth Revision	**Female sex, amplifier (dep. and anx.)** TV in boys: < 1 h/day, REF. 1, 2 hours/day, HR 1.09 (95%CI 0.78–1.53) 3–4 h/day, HR 1.12 (95% CI 0.79–1.60) 5h hours/day, HR 1.06 (95%CI 0.72–1.57) TV in girls: < 1 h/day, REF. 1, 2 hours/day, HR 0.80 (95%CI 0.63–1.00) (*p* < .05) 3–4 h/day, HR 0.81 (95%CI 0.63–1.04) 5h hours/day, HR 0.85 (95%CI 0.63–1.16) Computer/videogames in boys: < 1 h/day, REF. 1, 2 hours/day, HR 1.43 (95%CI 1.13–1.81) 3–4 h/day, HR 1.23 (95%CI 0.89–1.69) 5h hours/day, HR 1.35 (95%CI 0.93–1.96) Computer/videogames in girls: < 1 h/day, REF. 1, 2 hours/day, HR 1.18 (95%CI 1.00–1.42) (*p* < .05) 3–4 h/day, HR 1.31 (95%CI 1.00–1.74) (*p* < .05) 5h hours/day, HR 1.04 (95%CI 0.61–1.79) **TV viewing, buffer (dep. and anx.)** TV (entire sample): < 1 h/day, REF. 1, 2 hours/day, HR 0.89 (95%CI 0.74–1.08) 3–4 h/day, HR 0.91 (95%CI 0.74–1.11) 5h hours/day, HR 0.91 (95%CI 0.72–1.16) Computer/videogames (entire sample): < 1 h/day, REF. 1, 2 hours/day, HR 1.27 (95%CI 1.11–1.47) 3–4 h/day, HR 1.25 (95%CI 1.02–1.55) 5h hours/day, HR 1.23 (95%CI 0.92–1.64)	Moderate
Yan, 2017 [88]	2625 (15.1)	C	Self-reported average daily time spent TV viewing, videogame playing, studying on electronic devices, using social networking sites, or watching videos on school days and non-school days	Anxiety: The Middle School Student Mental Health Scale	**TV viewing, buffer (school days)** TV: Never, REF. < 1 h/day, β = − 0.023 (*p* = .210) 2–4 h/day, β = − 0.094 (*p* = .047) 4h hours/day, β = − 0.082 (*p* = .408) Videogames: Never, REF. < 1 h/day, β = − 0.027 (*p* = .199) 2–4 h/day, β = − 0.018 (*p* = .664) 4h hours/day, β = − 0.167 (*p* = .058) Studying on electronic devices: Never, REF. < 1 h/day, β = 0.012 (*p* = .542) 2–4 h/day, β = 0.062 (*p* = .036) 4h hours/day, β = 0.190 (*p* = .002) Social networking sites: Never, REF. < 1 h/day, β = 0.037 (*p* = .078) 2–4 h/day, β = 0.085 (*p* = .01) 4h hours/day, β = 0.106 (*p* = .025) Videos: Never, REF. < 1 h/day, β = 0.009 (*p* = .661) 2–4 h/day, β = 0.001 (*p* = .970) 4h hours/day, β = 0.013 (*p* = .846) **Social networking sites, amplifier (non-school days)** TV: Never, REF. < 1 h/day, β = − 0.033 (*p* = .131) 2–4 h/day, β = − 0.031 (*p* = .129) 4h hours/day, β = 0.034 (*p* = .208) Videogames: Never, REF. < 1 h/day, β = − 0.033 (*p* = .118) 2–4 h/day, β = − 0.028 (*p* = .192) 4h hours/day, β = − 0.010 (*p* = .711) Studying on electronic devices: Never, REF. < 1 h/day, β = 0.052 (*p* = .059) 2–4 h/day, β = 0.041 (*p* = .135) 4h hours/day, β = 0.060 (*p* = .055) Social networking sites: Never, REF. < 1 h/day, β = 0.072 (*p* = .009) 2–4 h/day, β = 0.087 (p = .002) 4h hours/day, β = 0.087 (*p* = .005) Videos: Never, REF. < 1 h/day, β = 0.001 (*p* = .975) 2–4 h/day, β = 0.001 (*p* = .964) 4h hours/day, β = 0.049 (*p* = .106)	Weak
Yang, 2013 [89]	10,829 (ages 10–12)	C	Self-reported average daily TV viewing and computer use (Internet games, non-Internet games, Internet for chatting, and “other” computer use)	Depression: select items from the SCL-90	**Female sex and TV viewing, amplifiers (sad or little interest in doing things)** TV in boys: OR 2.13 (95%CI 1.61–2.82) TV in girls: OR 3.54 (95%CI 2.59–4.85) Internet games in boys: OR 2.20 (95%CI 1.65–2.93) Internet games in girls: OR 2.85 (95%CI 1.70–4.78) Non-internet games in boys: OR 1.87 (95%CI 1.36–2.58) Non-internet games in girls: OR 2.25 (95%CI 1.28–3.97) Internet for chatting in boys: OR 2.0 (95%CI 1.42–2.84) Internet chatting in girls: OR 2.88 (95%CI 1.99–4.16) “Other” computer use in boys: OR 1.85 (95%CI 1.22–2.81) “Other” computer use in girls: OR 2.63 (95%CI 1.44–4.82) **Female sex and Internet games, amplifiers (cried easily or wanted to cry)** TV in boys: OR 2.37 (95%CI 1.66–3.40) TV in girls: OR 2.29 (95%CI 1.68–3.13) Internet games in boys: OR 1.79 (95%CI 1.20–2.65) Internet games in girls: OR 3.16 (95%CI 1.94–5.15) Non-internet games in boys: OR 1.82 (95%CI 1.19–2.79) Non-internet games in girls: OR 2.19 (95%CI 1.27–3.75) Internet for chatting in boys: OR 2.05 (95%CI 1.32–3.17) Internet chatting in girls: OR 2.60 (95%CI 1.83–3.69) “Other” computer use in boys: OR 2.67 (95%CI 1.64–4.36) “Other” computer use in girls: OR 2.91 (95%CI 1.63–5.17) **Female sex and other computer use, amplifiers (sad or blue)** TV in boys: OR 2.61 (95% 1.85–3.68) TV in girls: OR 2.52 (95%CI 1.79–3.55) Internet games in boys: OR 2.23 (95%CI 1.55–3.22) Internet games in girls: OR 2.76 (95%CI 1.62–4.68) Non-internet games in boys: OR 1.97 (95%CI 1.30–2.98) Non-internet games in girls: OR 2.70 (95%CI 1.52–4.78) Internet for chatting in boys: OR 2.26 (95%CI 1.48–3.46) Internet chatting in girls: OR 3.31 (95%CI 2.29–4.80) “Other” computer use in boys: OR 2.64 (95%CI 1.63–4.27) “Other” computer use in girls: OR 3.88 (95%CI 2.16–6.97) **Female sex and time on Internet for chatting, amplifiers (hopeless about future)** TV in boys: OR 2.91 (95% 2.09–4.06) TV in girls: OR 3.06 (95%CI 2.09–4.47) Internet games in boys: OR 2.70 (95%CI 1.90–3.83) Internet games in girls: OR 4.48 (95%CI 2.57–7.79) Non-internet games in boys: OR 3.13 (95%CI 2.18–4.47) Non-internet games in girls: OR 3.57 (95%CI 1.94–6.56) Internet for chatting in boys: OR 3.01 (95%CI 2.05–4.41) Internet chatting in girls: OR 5.41 (95%CI 3.68–7.96) “Other” computer use in boys: OR 2.92 (95%CI 1.84–4.63) “Other” computer use in girls: OR 4.89 (95%CI 2.62–9.12)	Weak
Ybarra, 2005 [90]	1501 (14.1)	C	Self-reported amount of general internet use	Depression: DSM-IV symptomology (major depression vs. minor depression vs, mild/no depression)	**Female sex, amplifier** In boys: Conditional OR 2.04 (0.90–4.65) (*p* = .09) In girls: Conditional OR 3.57 (95%CI 1.70–7.50) (*p* < .001)	Strong

Note: *C* Cross-sectional, *L* Longitudinal, *NS* nonsignificant, *SB* sedentary behavior, *OR* odds ratio, *HR* hazard ratio, *IRR* incidence rate ratio

### Sex (depressive symptoms: *n* = 43; anxiety symptoms: *n* = 20)

Forty-three studies tested sex as a moderator when investigating depressive symptom-screen-based SB associations, and of these, approximately half (*n* = 20) provided evidence that sex was a significant moderator. Fourteen (one among a clinical sample) of the 20 studies provided evidence for female sex as an amplifier of screen-based SB-depressive symptom associations [16, 29, 39, 40, 43, 52, 56, 62, 69, 81, 84, 87, 89, 90]. Furthermore, the majority (11 of the 14, one among a clinical sample) of these studies were conducted in large samples of over 1500 participants, including children and those in early- and mid- adolescence [29, 39, 40, 43, 56, 62, 81, 84, 87, 89, 90]. Only seven studies in adolescents found that female sex acted as a buffer of screen-based SB-depressive symptom associations [36, 46, 50, 67, 73, 82, 83]. Altogether, there is *inconsistent evidence* (46.5% of studies) of moderation of screen-based SB depressive symptom associations by sex*;* however, among the studies that found sex differences, 70.0% of studies identified female sex as an amplifier of associations.

Fewer studies (*n* = 20) investigated sex as a moderator of the relationship between screen-based SB and anxiety symptoms. Of the 20 studies, 14 studies did *not* provide evidence for moderation by sex [4, 23, 25, 26, 45, 47, 58, 62, 71, 72, 75, 80–82]. Of the six studies providing evidence for moderation by sex, five (one among a clinical sample and two with samples of over 10,000 participants) found female sex as an amplifier of screen-based SB-anxiety symptom associations [29, 43, 69, 70, 87]; while one study found that sedentary boys had more symptoms of anxiety compared to sedentary girls [67]. The summary result for moderation of screen-based SB-anxiety symptom associations by sex is *null* (30.0% of studies).

### Age (depressive symptoms: *n* = 6; anxiety symptoms: *n* = 1)

A cross-sectional study in 8029 students aged 10 to16 years old found that compared to the younger children in the sample, adolescents who met screen-time recommendations were less likely to develop depressive symptoms [60]. However, a longitudinal study of over 1700 adolescents found that screen-based SB was unrelated to symptoms of depression in those in early- and late- adolescence, however significant positive associations emerged in those in mid-adolescence [50]. Four studies (one among a clinical sample) with smaller sample sizes found that age was not a significant moderator of screen-based SB-depressive symptom associations [61, 72, 74, 76]. Thus, there is *inconsistent evidence* (33.3% of studies) for age as a moderator of screen-based SB-depressive symptom associations.

Only one investigation assessed age as a moderator of screen-based SB-anxiety symptom associations; this longitudinal and bi-directional study of over 500 young children did not provide evidence for age as a moderator (null interaction term) [72]. Because fewer than four studies assessed age as a moderator of screen-based SB-anxiety symptom associations, there is *inconsistent evidence* for moderation by age.

### Variables relating to country of origin/cultural factors (depressive symptoms: *n* = 2; anxiety symptoms: *n* = 0)

McHale et al. found that an aspect of Mexican cultural orientation, parental educational value, modified the relationship between TV viewing and depressive symptoms; symptoms of depression and TV viewing were only related to one another among children with fathers with low educational value in this cross-sectional study of 469 youth [65]. Additionally, a study conducted among Japanese and Czech children concluded that videogame playing reduced symptoms of depression in the Japanese children, while there were no associations found in the Czech sample [59]. Given the few available studies, there is *inconsistent evidence* for moderation by cultural factors.

### Physical activity (depressive symptoms: *n* = 6; anxiety symptoms: *n* = 3)

Three cross-sectional studies among adolescents found that moderate-to-vigorous PA or vigorous PA buffered screen-based SB-depressive symptom associations [30, 51, 55]. Contrarily, three studies among children and adolescents did not provide evidence for PA, including in the form of physical education classes and organized sports, as a significant moderator [26, 60, 83]. Taken together, there is *inconsistent evidence* (50.0% of studies) for PA as a moderator of screen-based SB-depressive symptom associations; however, among the studies with significant findings, 100.0% identified PA as a buffer of associations.

Only three studies assessed PA as a moderator of the relationship between screen-based SB and symptoms of anxiety. Two found that vigorous PA weakened screen-based SB-anxiety symptom associations [30, 51], while one did not provide evidence for leisure-time PA moderating screen-based SB-anxiety symptom associations [26]. Given the limited number of studies, there is *inconsistent evidence* for PA as a moderator of screen-based SB-anxiety symptom associations.

### Other potential moderators (depressive symptoms: *n* = 11; anxiety symptoms: *n* = 8)

Twelve studies investigated potential moderators that do not fall into the above categories. Three studies assessed how different peer/social factors (e.g., perceived friendship quality, in-person social interactions) may influence the strength of the relationship between screen-based SB and internalizing symptoms. For example, a recent longitudinal investigation revealed a three-way interaction between screen-based SB, sex, and social context in adolescents; girls who played videogames with peers were more likely to experience symptoms of anxiety, while boys who played videogames with peers were less likely to experience symptoms of anxiety [70]. In another study, there were no screen-based SB-internalizing symptom associations among youth with medium to high perceived friendship quality; while among children with low perceived friendship quality, instant messaging predicted fewer depressive symptoms and surfing the web was related to more depressive and anxiety symptoms in this subgroup [80]. The same study did not identify a three-way interaction between screen-based SB, sex, and perceived friendship quality among the sample [80]. Lastly, in a study of a nationally-representative sample, Twenge et al. found that social media use was only related to depressive outcomes among those with low in-person social interactions [84]. With the limited number of studies across different conceptualizations of the peer and social environment, there is *inconsistent evidence* for peer/social factors as moderators of screen-based SB-internalizing symptom associations.

Three studies investigated parental factors as potential moderators. Parental communication and parental involvement were not significant moderators of screen-based SB-internalizing symptom associations in studies among children and adolescents [49, 66]. Lastly, parental alcoholism did not moderate screen-based SB-depressive symptom associations, while it was an amplifier of the associations between surfing the web, playing videogames, and anxiety symptoms among a sample of 328 adolescents [69]. There is *inconsistent evidence* for parental factors moderating screen-based SB-internalizing symptom associations due to the limited number of available studies.

Four studies assessed potential moderation by a number of indicators and proxies for socioeconomic status including parental education, household income, and neighborhood-level socioeconomic characteristics, with each study providing no evidence of moderation [23, 34, 66, 72]. Taken together the summary result is *null* (0.0% of studies) for socioeconomic status as a moderator of screen-based SB-internalizing symptom associations.

Four studies looked at additional variables as potential moderators that do not fall into the above subcategories. Kim and Ahn found that neighborhood divorce rate weakened the positive longitudinal relationship between screen-based SB and depressive symptoms, whereas population size and education were not moderators [57]. In one longitudinal study of over 7000 pre-adolescent youth, investigators found that self-rated general health and pubertal status do not moderate screen-based SB-internalizing symptom associations [23]. Kleppang et al. assessed if the year of investigation (2001 vs. 2009) influenced the strength of screen-based SB-internalizing symptom associations and found no evidence of moderation [58]. Lastly, Internet use and sleep duration were independently associated with depressive symptoms, however the interaction between these two variables was not significant, and thus the strength of the association between Internet use and depressive symptoms did not vary by sleep duration [37].

### Type of screen-based sedentary behavior

Screen-based SB was conceptualized differently across studies, which may have influenced the strength of the observed associations with internalizing symptoms. Screen-based SB was typically defined as TV viewing, computer use, and videogame playing; resulting in investigators stratifying analyses by screen-type. Twenty-four studies provided evidence for differential associations by screen-type (e.g., TV viewing vs. computer use) when looking at screen-based SB in relation to depressive or anxiety symptoms; while eleven studies indicated that type of screen-based SB did not influence the strength of the observed associations. Taken together, there is *significant evidence* (68.6% of studies) of moderation by screen-type. Below, these individual studies are reviewed in more detail by screen-type.

### Comparison of associations of TV viewing with internalizing symptoms vs. another screen-based SB (depressive symptoms: *n* = 29; anxiety symptoms: *n* = 13)

More than half of the studies (*n* = 16, two among clinical samples) found that TV viewing was more weakly related to depressive symptoms compared to at least one other form of screen-based SB, such as computer use and videogame playing [35, 40–42, 50, 54, 61–64, 67, 68, 76, 84, 86, 87]. Three (one among a clinical sample) studies determined that TV viewing was related to depressive symptoms, with similar effect estimates compared to other electronic use investigated [35, 42, 77]. Five studies indicated that TV viewing was more strongly related to depressive symptoms compared to at least one other form of screen-based SB [28, 31, 33, 44, 73] and seven studies (one among a clinical sample) provided evidence that all forms of screen-based SB (including TV) were unrelated to depressive symptoms [24, 28, 47, 69, 71, 72, 74]. Lastly, Yang et al. found that TV viewing among girls was more strongly related to the depressive symptom of feeling sad or having little interest in doing things compared to other screen-types [89]; however, this study has also demonstrated that TV viewing was more weakly related to other symptoms of depression such as crying easily or feeling hopeless about the future, compared to other forms of screen-based SB [89].

Thirteen studies (two among clinical samples) examined TV viewing-anxiety symptom associations among youth. A majority (*n* = 9) of studies found that compared to at least one other type of screen-based SB, TV viewing was not as strongly related to symptoms of anxiety [42, 62–64, 67, 69, 86–88]. One study indicated that TV viewing, along with other forms of screen-based SB, were each related to symptoms of anxiety with similar magnitudes (cross-sectionally only) [42]. Lastly, four investigations found that all forms of screen-based SB (including TV) were unrelated to anxiety symptoms [24, 47, 71, 72].

### Comparison of associations of computer use/internet use with internalizing symptoms vs. another screen-based SB (depressive symptoms: *n* = 29; anxiety symptoms: *n* = 12)

Of the 29 studies that assessed the relationship between computer use and depressive symptoms, five determined that computer use was more weakly associated with depressive symptoms compared to at least one other form of screen-based SB [35, 44, 50, 67, 84]; and two studies found a protective association between computer use and depressive symptoms while simultaneously demonstrating either null or positive associations between another form of screen-based SB and depressive symptoms [33, 64]. Thirteen studies (two among clinical samples) concluded that computer usage was more strongly related to depressive symptoms compared at least one other form of screen-based SB [31, 40–42, 54, 61–63, 68, 76, 86, 87, 89]. Cross-sectionally, Bickham et al. did not find associations between any form screen-based SB and depressive symptoms, however longitudinally, TV viewing and mobile phone use were positively associated with depressive symptoms while computer and videogame use remained unrelated to depressive symptoms [28]. Three studies (one among a clinical sample) concluded that computer use was associated with depressive symptoms, with similar effect estimates compared to the other forms of screen-based SB investigated [35, 42, 77]. Finally, seven studies (one among a clinical sample) found that engagement in each screen-type (including computer use) was unrelated to depressive symptoms [24, 69, 71, 72, 74, 85, 92].

Four studies (two among clinical samples) found that computer use was positively associated with symptoms of anxiety, and that computer use was more strongly related to symptoms of anxiety than other forms of screen-based SB [62, 69, 86, 87]; whereas one study found that computer use was protective against symptoms of anxiety [64]. Only two studies found that computer use was more weakly associated with symptoms of anxiety compared to at least one other form of screen-based SB [63, 67]. Cross-sectionally, Gopinath et al. found that each type of screen-based SB (including computer use) was similarly related to symptoms of anxiety, whereas longitudinally, computer use was more strongly related to symptoms of anxiety compared to TV viewing [42]. Lastly, four studies found that each form of screen-based SB assessed was unrelated to symptoms of anxiety [24, 71, 72, 92].

### Comparisons of associations of videogame playing with internalizing symptoms vs. another screen-based SB (depressive symptoms: *n* = 21; anxiety symptoms: *n* = 12)

Eleven of 21 studies (two among clinical samples) concluded that videogames were more strongly related to depressive symptoms compared to at least one other form of screen-based SB [41, 42, 61–64, 67, 68, 76, 86, 87]; while four studies found that videogame playing was more weakly related to symptoms of depression compared to at least one other screen-type [28, 35, 50, 73]. Further, one study found that videogames were protective against symptoms of depression [31]. Videogame playing, along with other forms of screen-based SB were each similarly positively associated with depressive symptoms in two studies [35, 42]. Lastly, six studies (one among a clinical sample) found that videogame playing (along with all other screen-types) was not related to depressive symptoms [24, 28, 69, 71, 72, 74].

Seven of twelve studies (two among clinical samples) found a stronger association between videogame playing and symptoms of anxiety as compared to another form of screen-based SB [42, 62–64, 67, 86, 87]. Alternatively, one study indicated that videogames were more weakly associated with anxiety symptoms as compared to other screen-types [88]. Ohannessian found a positive association between videogames and symptoms of anxiety in girls, but a negative association between these two variables in boys [69]. Lastly, one found that engagement in all screen-types were similarly positively associated with anxiety symptoms [42], while three studies found that each screen-based SB was similarly unrelated to anxiety symptoms [24, 71, 72].

### Comparisons of associations of other screen-based sedentary behaviors with internalizing symptoms (depressive symptoms: *n* = 10; anxiety symptoms: *n* = 4)

In addition to the three main types of screen-based SB (TV viewing, computer use, and videogame playing), there were some investigations of other screen-types in relation to internalizing symptoms. Six studies assessed how texting/mobile phone use related to depressive symptoms; and of these, only one study found that texting/mobile phone use was more strongly related to depressive symptoms compared to other screen-types longitudinally [28]. Alternatively, two studies found that texting/mobile phone use had weaker associations with depressive symptoms compared to other forms of screen-based SB [61, 64]; while three studies concluded that texting/mobile phones were similarly unrelated to depressive symptoms compared to other forms of screen-based SB [69, 72, 85]. Social media was also a screen-based SB that was investigated in two studies; both demonstrated that social media use was more strongly related to depressive symptoms compared to other forms of screen-based SB [50, 84]. In another study, tablet use on weekdays was more strongly related to depressive symptoms compared to other screen-types, however this pattern did not emerge on weekend days, when all screen-based SB were similarly positively associated with depressive symptoms [35]. Lastly, Primack et al. found that watching videos was unrelated to depressive symptoms, compared to TV viewing which was positively associated with depressive symptoms in a longitudinal study of over 4000 participants [73].

Only four studies investigated the relationship between other types of screen-based SB and symptoms of anxiety. Specifically, two studies found that texting was more weakly related to symptoms of anxiety as compared to at least one other form of screen-based SB [64, 69]. On the other hand, one study found that mobile phone use was similarly bi-directionally unrelated to symptoms of anxiety compared to other screen-types [72]. Lastly, studying on electronic devices and going on social networking sites on schooldays, specifically, were more strongly related to symptoms of anxiety compared to other forms of screen-based SB, such as watching videos, in a study of over 2600 adolescents [88]. On non-school days, however, only social networking sites were related to symptoms of anxiety in this sample [88].

### Sensitivity analyses among studies of high methodological quality (depressive symptoms: *n* = 6; anxiety symptoms: *n* = 1)

Sensitivity analyses of the six studies with high methodological quality were conducted to gain a better understanding of findings across studies that were rated as the least likely to be subject to biases; five were of depressive symptoms [33, 53, 57, 73, 90] and one was of depressive and anxiety symptoms [30].

Sex was tested as a moderator in three studies, with mixed findings [53, 73, 90]. Stratified analyses among approximately 1500 adolescents indicated that Internet use was related to depressive symptoms among girls, but not boys [90]. However, a later longitudinal study of over 4000 adolescents found that female sex was a buffer of screen-based SB-depressive symptom associations via a statistical test of interaction [73]. Lastly, a study of the association between clusters of energy balance behaviors and depressive symptoms found that those within the clusters encompassing the highest levels of screen-based SB had the greatest level of depressive symptoms, regardless of sex [53].

The influence of screen-type on screen-based SB-depressive symptom associations was investigated in two studies, and differences by screen-type emerged in both [33, 73]. In a longitudinal study of over 10,000 adolescents, investigators found that Internet games were protective of subsequent depressive symptoms, while TV viewing was unrelated to depressive symptoms [33]. On the other hand, Primack et al. found that among the screen-types investigated (TV viewing, videos, and videogame playing), only TV viewing was longitudinally related to increased odds of depressive symptoms [73].

In a longitudinal study of middle-schoolers, Kim et al. found that among various neighborhood characteristics, including divorce rate, population size, and education level, only neighborhood divorce rate influenced the strength of the association between videogame playing and subsequent depressive symptoms [57]. Neighborhoods with a higher neighborhood divorce rate buffered the association between videogame playing and depressive symptoms, as indicated by a significant test for interaction [57]. Lastly, a study of over 5000 adolescents demonstrated that vigorous PA buffers the association between screen-based SB, depressive symptoms, and anxiety symptoms [30].

## Discussion

The aim of this paper was to summarize the evidence for potential moderators of screen-based SB and internalizing symptom associations to better understand the heterogeneity of previous study findings, identify at-risk populations, and to pose future research directions for the field. Screen-type most consistently influenced the strength of the association between screen-based SB and internalizing symptoms. However, the evidence for symptoms of anxiety, specifically, is more limited, and therefore findings should be interpreted with caution. Currently, the literature provides *inconsistent* evidence for moderating effects by sex, age, cultural characteristics, PA, peer factors (e.g., friendship quality and social context), and parental factors (e.g., parental communication). Further, there is no evidence of moderation by socioeconomic status.

### Synthesis of findings and implications

Our results indicate that screen-type influences the strength of the screen-based SB-depressive symptoms relationship; TV viewing appears less likely to be associated with depressive symptoms, compared to computer use and videogame playing. Screen-specific associations highlight that psychosocial mechanisms, which only occur when one is engaged in certain forms of screen-based SB, are likely to explain the link between screen time and internalizing symptoms. For example, being a victim of cyber-bullying may contribute to the positive association between computer/Internet use, specifically, and depressive symptoms [93, 94]. Further, the online environment may be especially hostile for adolescent girls, who report being exposed to more unintentional negative online content (sexual content, slander) compared to boys [95]. Additionally, passive social media use, the monitoring of others’ lives by viewing the content of their profiles, may increase susceptibility to internalizing symptoms [96, 97]; passive social media use may be particularly detrimental because it increases one’s feelings of inferiority via upward social comparison and can increase perceived loneliness [98]. On the other hand, utilizing the computer for instrumental reasons, such for school work or email, is not associated with increased symptoms of depression in youth [82]. Taken together, computer/Internet use may be more worse for the emotional well-being of youth compared to other forms of screen-based SB when it is utilized for specific purposes.

Similarly, only certain forms of videogaming may have negative consequences for youth. A study among children found that violent videogames were correlated with depressive symptoms, while videogames without violent content were unrelated to depressive outcomes [99]. Frequent and competitive video gaming (playing against others) may also pose negative implications for psychosocial well-being [100]. Conversely, other work has shown that videogaming may have a positive influence on development [101]. Playing casual videogames—ones that are fun, easy to learn, and readily accessible (i.e. *Bejeweled II*)—can improve mood and promote relaxation [102, 103]; further, a review and meta-analysis provides evidence for the efficacy of *reducing* internalizing symptoms with certain types of videogames, even among clinical samples [104]. Thus, emotional influence of videogames may depend on the content, type, and purpose of the videogame play. Taken together with our findings that computer and videogame use are more likely to be associated with depressive symptoms compared to television viewing, interventions aimed at reducing symptoms of depression via reducing sedentary time may most effective if they targeted computer use and videogame playing, specifically. However, future research should attempt to gain a better understanding of how specific behaviors within computer and videogame use (e.g., passive vs. active social media use) relate to internalizing symptoms prior to the development of intervention strategies, given that not all types and content of computer use and videogame play may have emotional consequences.

Sex was among the most commonly-studied moderators of screen-based SB-internalizing symptom associations; our review revealed inconsistent evidence for moderation by sex. Inconsistencies in the evidence for sex as a moderator may be due unexplored three-way interactions between sex, screen-based SB (or internalizing symptoms), and another third variable, for example age. It is conceivable that sex may be an important moderator of screen-based SB-internalizing symptom associations only during more vulnerable periods in development when sex differences in activity and internalizing symptom levels become more apparent [105–108], and not during earlier or later stages in development. However, more investigations of the potential three-way interaction between sex, screen-based SB (or internalizing symptoms), and age are needed.

Across studies that did find significant moderation by sex, it appears that sedentary girls, but not boys, may be more susceptible to depressive symptoms. Girls have higher rates of depression which may, in part, be a result of biological and cognitive factors such as hormonal changes [109] and ruminative coping [110]. On a behavioral level, girls may prefer more unhealthy forms of screen-based SB compared to boys; a recent study found that girls were significantly more likely than boys to use social networking sites for more than two hours daily [111]. The use of social networking sites may be particularly deleterious because they can increase one’s susceptibility to depressive symptoms via upward social comparison [112]. On a psychosocial level, boys and girls tend to engage in screen-based SB in similar social contexts; youth oftentimes engage in screen-based SB with others, including with friends and family members [113, 114]. However, the social context of screen-based SB may interact with sex such that screen-based SB with friends may be protective against internalizing symptoms among boys, but not girls [70]. Although sedentary girls appear to be at greater risk compared to sedentary boys, a more nuanced understanding mechanisms underlying the interaction between sex and screen-based SB on internalizing symptoms is still needed prior to the development of intervention strategies. Future research should investigate potential sex differences in (1) preferred forms of screen-based SB, (2) psychosocial behaviors (e.g., social comparison) while engaging in screen-based SB, and (3) social context of screen-based SB, to further our understanding sedentary girls’ potential vulnerabilities. Once this is accomplished, future interventions aimed at decoupling screen-based SB and internalizing symptoms may be tailored towards specific screen-based SB among at-risk groups, increasing the likelihood of intervention success.

The few studies investigating PA, including organized sports, as a moderator suggest that it may weaken screen-based SB-internalizing symptom associations. Participation in sports is associated with fewer symptoms of depression and anxiety in some children [115], which may be explained by increases in self-esteem and social support [116]. However, participation in organized sports may not be an appropriate intervention strategy for all youth; students identifying as lesbian, gay, bisexual, transgender, and queer are commonly bullied in the school-based athletic setting (physical education class and afterschool sports) [117]. Structured aerobic PA interventions, consisting of activities such as cycling and jogging, have also demonstrated efficacy for reducing symptoms of depression and anxiety, even among clinical populations [118, 119]. However, a notable limitation of structured PA interventions is their lack of sustainability in the real-world setting. Adherence to PA programs steadily decreases over time, especially among those with depressive symptoms [120]. Therefore, future investigations should test additional, less-understood, more feasible, and enjoyable forms PA as a moderator; as certain types of PA may be vital intervention strategies for decoupling screen-based SB and their associated emotional health consequences in the real-world setting.

### Suggestions for future research

While continuing to test variables such as sex, types of PA, and content with each type of screen-based SB as potential moderators is pertinent to future research, it should be recognized that the current body of literature contains limitations that must be addressed with future, high quality investigations. First, more than half of the studies in this review were cross-sectional (*n* = 46), which prevents our ability to draw causal conclusions and limits our understanding of the directionality of associations. Future research should focus on gaining a better understanding of the directionality of screen-based SB-internalizing symptom associations as recent studies indicate that screen-based SB and internalizing symptoms may have a bi-directional relationship [121, 122]. However, additional studies with longitudinal and experimental designs are needed to assist with determining the potential for causal and bi-directional associations between screen-based SB and internalizing symptoms. Future studies aimed at pinpointing the directionality of associations will ultimately strengthen our ability to identify critical intervention points for decoupling screen-based SB and internalizing symptoms.

Second, a majority of the studies in the present review relied on paper-and-pencil, retrospective self-reports of engagement in screen-based SB. A combination of device-based measures (e.g., inclinometers, accelerometers) and real-time self-reports (e.g., ecological momentary assessment) should be used to limit recall bias and provide more detailed information on types and contexts of screen-based SB, as this review suggests these factors can influence associations with internalizing symptoms among youth [123].

Third, the body of evidence for depressive symptoms is much larger than that for anxiety symptoms. Anxiety symptoms are likely related to screen-based SB, but the moderating factors of this association remain unclear, due to the limited evidence available. One potential mechanism for this trend is that the physiologic hyperarousal associated with anxiety symptoms may reduce the likelihood of being influenced by moderators as compared to depressive symptoms, where hyperarousal is not present [124]. Similarly, many studies used measures that combined depressive and anxiety symptoms within the same subscale; making it difficult to interpret if results are applicable to depressive symptoms only, anxiety symptoms only, or both. Recent evidence indicates that differential associations appear between screen-based SB and symptoms of various forms of anxiety; specifically, screen-based SB may be related to symptoms of generalized anxiety and social phobia, but not panic disorder [125]. Therefore, future research should be directed toward gaining a better understanding of anxiety symptoms and screen-based SB in addition to depressive symptoms, by using separate scales to quantify symptoms of each internalizing disorder.

Lastly, a majority of the evidence for sex as a moderator is based on stratified analyses without statistical tests for interaction, therefore results must be interpreted with caution. Future research should rely only on formal statistical tests for interaction when assessing moderation. Timely interventions for decoupling screen-based SB and depressive and anxiety symptoms are critically needed, as the prevalence of certain (and potentially more deleterious) forms screen-based SB continues to rise [126, 127]; the abovementioned suggestions for future research may be pivotal in designing and optimizing future intervention strategies.

### Strengths and limitations

A strength of the present review is the systematic search strategy used, which yielded a considerable number of studies included in the present review; however, there is the possibility that our search strategy may have missed relevant studies on the topic. Another limitation of this review is that only published articles written in English were included, index terms were not used, and a “grey literature” search was not included; therefore, our evidence may be biased towards positive results due to publication bias. Furthermore, studies that included screen-based SB in addition to non-screen-based SB (e.g., reading, homework) as a composite variable were excluded. Similarly, studies examining the nuances within the screen-based SB construct, such as content, weren’t systematically reviewed in the present article.

## Conclusions

This review summarizes moderators of the screen-based SB-internalizing symptom associations, discusses potential mechanistic explanations, and poses directions for future research. There is consistent evidence that screen-type influences the strength of the association between screen-based SB and internalizing symptoms. Less consistent evidence is available for female sex as an amplifier and physical activity as a buffer, therefore more research is needed on these factors to identify the possibility of vulnerable populations and tailored intervention strategies. Additionally, more evidence is needed for anxiety symptoms in particular. Gaining a thorough understanding of these complex relationships will lead to effective intervention strategies for improving the emotional and physical health of youth to ultimately prevent morbidity and mortality later in life.

## Data Availability

Not applicable.

## Abbreviations

SB: Sedentary behavior
PA: Physical activity
